# Apoptosis repressor with caspase recruitment domain (ARC) promotes bone regeneration of bone marrow-derived mesenchymal stem cells by activating Fgf-2/PI3K/Akt signaling

**DOI:** 10.1186/s13287-021-02253-5

**Published:** 2021-03-16

**Authors:** Longwei Hu, Yang Wang, Hongya Pan, Kathreena Kadir, Jin Wen, Siyi Li, Chenping Zhang

**Affiliations:** 1 Department of Oral & Maxillofacial-Head & Neck Oncology, Ninth People’s Hospital, College of Stomatology, Shanghai Jiao Tong University School of Medicine; National Clinical Research Center for Oral Diseases; Shanghai Key Laboratory of Stomatology& Shanghai Research Institute of Stomatology, Shanghai, 200011 People’s Republic of China; 2 Linno Pharmaceuticals Inc., Shanghai, 200011 People’s Republic of China; 3 Department of Oral & Maxillofacial Clinical Sciences, Faculty of Dentistry, University of Malaya, 50603 Kuala Lumpur, Malaysia; 4 Department of Prosthodontics, Ninth People’s Hospital affiliated to Shanghai Jiao Tong University, School of Medicine, Shanghai, 200011 People’s Republic of China

**Keywords:** ARC, BMSCs, Osteogenic differentiation, Fgf-2

## Abstract

**Objectives:**

This study aims to investigate whether apoptosis repressor with caspase recruitment domain (ARC) could promote survival and enhance osteogenic differentiation of bone marrow-derived mesenchymal stem cells (BMSCs).

**Materials and methods:**

The lentivirus transfection method was used to establish ARC-overexpressing BMSCs. The CCK-8 method was used to detect cell proliferation. The BD Pharmingen™ APC Annexin V Apoptosis Detection kit was used to detect cell apoptosis. The osteogenic capacity was investigated by OCN immunofluorescence staining, ALP analysis, ARS assays, and RT-PCR analysis. Cells were seeded into calcium phosphate cement (CPC) scaffolds and then inserted subcutaneously into nude mice and the defect area of the rat calvarium. Histological analysis was conducted to evaluate the in vivo cell apoptosis and new bone formation of the ARC-overexpressing BMSCs. RNA-seq was used to detect the possible mechanism of the effect of ARC on BMSCs.

**Results:**

ARC promoted BMSC proliferation and inhibited cell apoptosis. ARC enhanced BMSC osteogenic differentiation in vitro. An in vivo study revealed that ARC can inhibit BMSC apoptosis and increase new bone formation. ARC regulates BMSCs mainly by activating the Fgf-2/PI3K/Akt pathway.

**Conclusions:**

The present study suggests that ARC is a powerful agent for promoting bone regeneration of BMSCs and provides a promising method for bone tissue engineering.

## Introduction

Bone defects caused by tumors, trauma, inflammation, or congenital deformity greatly impact patients’ quality of life [1]. Autogenous bone grafts are considered the gold standard for the treatment of these defects. However, this strategy is limited by donor site morbidity, insufficient bone volume, and incomplete integration into the defect [2]. Bone tissue engineering strategies can provide regenerative tissues to repair bone defects without the aforementioned limitations. In these approaches, bone-forming cells are often used in combination with biomaterial scaffolds and growth factors [3]. Bone marrow-derived mesenchymal stem cells (BMSCs) have a powerful proliferative ability and multilineage differentiation potential, are easily available, and are crucial for bone remodeling and repair. BMSCs have been shown to successfully enhance bone repair and thus are widely used in bone tissue engineering [4, 5]. BMSCs can be seeded into biomaterial scaffolds along with incorporated growth factors or genes, thus promoting their differentiation into osteoblasts that are capable of producing new bone matrix. Nevertheless, these implanted cells encounter hypoxia or even anoxia within the implant after they are implanted into the defect area, which can lead to low cell survival and may even cause failure of the implant [6, 7]. Hence, exploring an agent that can both increase the ability of cells to protect against hypoxia-induced apoptosis and promote osteogenic differentiation may be a promising method to achieve the success of these regenerative implants.

Apoptosis repressor with caspase recruitment domain (ARC), which is also termed NOL3, is a highly potent and multifunctional inhibitor of apoptosis [8]. This molecule is physiologically expressed in terminally differentiated cells, including cardiomyocytes, neurons, and skeletal muscle cells [9]. ARC can inhibit both intrinsic and extrinsic apoptosis and is able to protect cells from hypoxia-induced cell apoptosis and death [10, 11]. It was reported that cardiomyocytes isolated from neonatal ARC transgenic mice showed significant resistance to hypoxia and that ARC can protect cardiomyocytes from hypoxia-induced cell death by regulating downstream Drbp1 and pMe(2)GlyDH [12]. It was also found that downregulation of the ARC protein in hippocampal neurons may eventually lead to hypoxia-induced neuronal death [13]. In addition to the ability to inhibit hypoxia-induced cell death, our previous study confirmed that ARC can promote human osteoblast osteogenic differentiation [14].

Thus, ARC may be a powerful agent to enhance new bone formation in vivo after transduction into BMSCs given its ability to prevent hypoxia-induced cell death and its potential to promote osteogenic differentiation of osteoblast cells. In this study, the effects of ARC on BMSCs were detected. We established ARC-overexpressing BMSCs, detected whether ARC could inhibit BMSC apoptosis and enhance osteogenic differentiation in vitro, and evaluated whether ARC could promote new bone formation by BMSCs in vivo. Then, we further explored the possible mechanism.

## Methods

### BMSC isolation and culture

BMSCs from rats were isolated and cultured according to previously described protocols [15]. Briefly, the femurs and tibias were bluntly dissected aseptically after the rats were sacrificed by cervical dislocation. The bone marrow was then flushed quickly with DMEM containing 10% fetal bovine serum (FBS, HyClone, Logan UT, USA), 100 U/mL penicillin, 100 U/mL streptomycin, and 200 U/mL heparin (Sigma, St Louis, MO, USA). The primary cells were resuspended in DMEM after centrifugation at 2000 rpm for 10 min and then cultured at 37 °C in a humidified atmosphere of 5% CO_2_. Twenty-four hours later, the nonadherent cells were removed. The DMEM was refreshed every 3 days, and the cells at passage 2 or 3 were used for future experiments. Each experiment was repeated 3 times using 3 different BMSCs strains isolated from 3 rats. This study was conducted under the approval of the Animal Research Ethical Committee of the Ninth People’s Hospital Affiliated with Shanghai Jiao Tong University School of Medicine.

### Lentiviral packaging and BMSC transduction

Human embryonic kidney 293T cells (HEK293T) were used for lentiviral packing. HEK293T cells was provided by Genechem Co., Ltd. (Shanghai, China) and originally purchased from American Type Culture Collection (ATCC, Manassas, VA, CRL-3216). The cells were cultured in 10 cm dishes for 2–3 days until they reached 90–95% confluency. The recombinant virus plasmid pLV-nol3-EGFP, which encodes the full-length nol3, and the control vector pLV-EGFP, together with packaging plasmids (pLP1, pLP2, and pLP/VSVG), were cotransfected into HEK293T cells using Lipofectamine™ 2000 (all from GeneChem Co., Ltd., Shanghai, China). After 48 h of transduction, the lentiviral particles contained in the supernatant of 293T cells were harvested and then concentrated by passing through a 0.45-μm filter. The concentrated solutions were then added to cultured BMSCs at a variety of multiplicities of infections ranging from 0 to 100. After 72 h, the transduction efficiency was assessed via fluorescence microscopy and qRT-PCR.

### Analysis of cell proliferation

Cell Counting Kit-8 (CCK-8, Dojindo, Kumamoto, Japan) was used to detect cell proliferation. Cells were seeded on 96-well plates at a density of 1.5 × 10^3^ cells/well, and the proliferation was detected for 72 consecutive hours. Ten microliters of CCK-8 reagent with 100 μl of DMEM was added to each well and then incubated at 37 °C for 2 h. The absorbance was then measured with a microplate reader (Bio-Tek, Winooski, VT, USA) at a wavelength of 490 nm according to the manufacturer’s instructions.

### Analysis of cell apoptosis

Cells were seeded on 6-well plates at a density of 1 × 10^5^ cells/well and then cultured for 72 h. The supernatant and adherent cells were collected and then centrifuged at 1500 rpm for 10 min at 4 °C. For detection of cell apoptosis, the BD Pharmingen™ APC Annexin V Apoptosis Detection kit (BD Biosciences, Franklin Lakes, NJ, USA) was used according to the manufacturer’s protocol. Briefly, the cells were washed with cold PBS twice and resuspended in 500 μL of binding buffer. Then, 5 μL of Annexin V-APC solution was added to the solution and incubated in the dark for 15 min at 37 °C. Finally, 1 μL of energy-coupled dye (ECD-A) solution was added for the subsequent flow cytometry for cell apoptosis analysis.

### Immunofluorescence staining

Cells were seeded at a density of 2.5 × 10^4^ cells/well in 24-well plates. After the corresponding treatment, the cells were washed with PBS, fixed with 4% paraformaldehyde for 15 min at 4 °C, and then permeabilized with 0.5% PBST (PBS containing 0.5% Triton X-100). Next, the cells were blocked with 5% bovine serum albumin for 1 h at room temperature and then incubated with anti-OCN antibodies for 1 h at 37 °C. The cells were washed twice with PBST and incubated with secondary antibody for 1 h at room temperature. After two washes with PBST, the nuclei were stained with DAPI (Invitrogen) for 5 min. Photographs were visualized under a light microscope (Olympus Corporation, Tokyo, Japan).

### Alkaline phosphatase activity (ALP) detection and Alizarin red staining (ARS)

Cells were seeded at a density of 2.5 × 10^4^ cells/well in 24-well plates and cultured for 7 days. For ALP staining, the cells were washed twice with PBS and fixed with paraformaldehyde for 15 min at 4 °C. Then, paraformaldehyde was removed, and the cells were washed again twice with PBS. Then, BCIP/NBT solution was added to each well and incubated in the dark for 30 min. All procedures were performed according to the protocols of the ALP staining kit (Beyotime Institute of Biotechnology, Shanghai, China). For ALP activity detection, a BCA protein assay kit (Beyotime Institute of Biotechnology, Shanghai, China) was used, and the ALP levels were normalized to the total protein content and are described as the percentage of total protein.

For ARS measurements, the cells were cultured in DMEM for 21 days, washed twice with PBS, and then fixed with paraformaldehyde for 15 min at 4 °C. The paraformaldehyde was removed, and the cells were washed twice again with PBS. Then, the cells were stained with 40 mM ARS for 20 min at room temperature. The stain was desorbed with 10% cetylpyridinium chloride (Sigma) for 1 h, and the solution was collected and equally distributed on a 96-well plate. Finally, a spectrophotometer (Bio-Tek) was used to determine the concentration of Alizarin red at 590 nm wavelength.

### Real-time PCR analysis of gene expression

TRIzol reagent (Invitrogen; Thermo Fisher Scientific, Inc.) was used to extract total cellular RNA. Reverse transcription was performed using the PrimeScript RT reagent kit (TaKaRa Bio, Inc., Otsu, Japan). Gene-specific primers were synthesized commercially (Shengong Co., Ltd., Shanghai, China), and their sequences are listed in Table 1. In one reaction, 10 μl of SYBR Premix Ex Taq kit (TaKaRa Bio, Inc.) was used to amplify 1 μl of cDNA (mixed with 8 μl of distilled water and 0.5 μl of each primer). The Bio-Rad iQ5 real-time PCR system (Bio-Rad Laboratories, Inc., Hercules, CA, USA) was then used to detect gene expression. All relative gene expression values were normalized to β-actin based on the 2^−ΔΔ^Cq method.

**Table 1:** Sequences of the primers used for reverse transcription quantitative polymerase chain reaction

Genes	Forward primer	Reverse primer
Actin	TGAAGTGTGACGTGGACATC	GGAGGAGCAATGATCTTGAT
ARC	ACAGCCTTGGGAAGTGAGAC	GTCCAGAGAGAAGCCAGACAA
ALP	TCAAGCCAAACACAAACAGC	GGAGCCACAATCCAGTCATT
OCN	GCGAGACATCAAGGAGAAGC	CCAATAAAGGAAGGCTGGAA
Runx2	GAAGAGGAGGAGGAAGAAGAGG	TCCATAGCCCAGTGTTGTAGC
Fgf-2	CAACACTTACCGGTCACGGA	CCCCGTTTTGGATCCGAGTT

### Western blot analysis

For Western blot analysis, cells were lysed with RIPA buffer supplemented with a protease inhibitor cocktail after culture for 7 days. The obtained protein concentrations were measured by a BCA protein assay kit (Beyotime, Shanghai) according to the manufacturer’s instructions, and then equal amounts of protein from different samples were separated by sodium dodecyl sulfate-polyacrylamide gel electrophoresis and transferred to polyvinylidene difluoride membranes. Membranes were incubated overnight at 4 °C with the following primary antibodies: rabbit anti-rat Akt, rabbit anti-rat pi-Akt, and rabbit anti-rat pi-PI3K (1:1000, Cell Signaling Technology, Inc.). Finally, the membranes were visualized with horseradish peroxidase-conjugated goat anti-rabbit using ECL Plus reagents and an UVItec Alliance 4.7 gel imaging system.

### Ectopic new bone formation and cell apoptosis analysis in nude mice

Cells were seeded in calcium phosphate cement (CPC) scaffolds after being cultured for seven days under normoxic conditions. A total of 14 constructs were divided into two groups: group A, CPC/BMSC-CON *n =* 7; group B, CPC/BMSC-NOL3 *n* = 7. Then, they were inserted into the subcutaneous space of nude mice. Ten weeks later, the mice were sacrificed by an overdose injection of ketamine, and the implanted specimens were harvested. After 4% paraformaldehyde fixation, the specimens were decalcified in 20% EDTA (pH = 7.4) for 10 days followed by paraffin-embedding, sectioning and staining with HE (hematoxylin and eosin). Immunohistochemical staining was carried out with a primary antibody against OCN (Cell Signaling Technology, Inc.). Cell apoptosis was detected by using a TUNEL kit (Cell Signaling Technology, Inc.). Tissue slides were visualized under a light microscope (Olympus Corporation, Tokyo, Japan), and Image-Pro 6.0 software (Media Cybernetics, Inc., Rockville, MD, USA) was used to perform histomorphological analysis. New bone formation was defined as the percentage of observed new bone area in the entire implant.

### Surgical procedures

Cells were loaded in the CPC scaffold at a density of 2.0 × 10^5^ cells/ml before being implanted into the defect area of the rat calvarium. The scaffold was shaped into a cylinder (5 mm× 2 mm^3^) and had an average pore size of 400 mm ± 50 mm and 75% porosity. A rat calvarial defect model was created to evaluate the in vivo new bone formation of BMSCs. The surgical procedures were performed according to previously described protocols [16]. Briefly, the rats were placed in a prone position after anaesthetization by intraperitoneal injection of ketamine. A 1.5-cm-long sagittal incision was made on the scalp, and then the calvarium was exposed by blunt dissection. Then, defects 5 mm in diameter were created bilaterally by using a trephine bur (Fine Science Tools, Foster City, CA, USA). The BMSC/CPC constructs were inserted into the defective area, and the wound was tightly closed. The rats were housed in ventilated rooms with access to sterilized water and food after the surgery. Nine 12-week-old male Sprague Dawley rats (SD rats) were randomly divided into three groups: (1) CPC with BMSC-CON (*n* = 3), (2) CPC with BMSC-NOL3 (*n* = 3), and (3) CPC alone (*n* = 3).

### Sequential fluorescence labeling

Polychrome sequential fluorescence labeling was used to characterize the mineralizing tissues. At 2, 4, and 6 weeks after the operation, different fluorochromes were injected intraperitoneally in the following sequence: tetracycline hydrochloride (25 mg/kg, Sigma Aldrich), Alizarin red S (30 mg/kg, Sigma Aldrich, USA), and calcein (20 mg/kg, Sigma Aldrich).

### Sample preparation

The rats were sacrificed by an overdose injection of ketamine 8 weeks after surgery. The calvarium with the implants was harvested and fixed in 10% paraformaldehyde for the following histological evaluation.

### Histological evaluation

Three specimens from each group were dehydrated in ascending concentrations of ethanol and then embedded in polymethylmethacrylate (PMMA). The specimens were cut into 150 mm thick sections by using a saw microtome (Leica, Hamburg, Germany) and polished to a final thickness of approximately 40 mm. A confocal laser scanning microscope (Leica TCS, Sp2 AOBS) was used for fluorescence labeling detection. The excitation/emission wavelengths used to observe fluorochromes were 405/580 nm, 543/617 nm, and 488/580 nm for tetracycline (yellow), Alizarin (red), and calcein (green), respectively. Then, the sections were stained with Van Gieson’s picro fuchsin for mineralized bone tissue visualization. The images were acquired by using a fluorescence microscope (Olympus, Japan). Histomorphometric analysis was conducted by Image-Pro Plus 6.0 software. New bone formation was defined as the percentage of observed new bone area in the entire implant.

### RNA-seq and bioinformatics

Three paired samples were used for RNA sequencing and bioinformatic analysis. Total RNA was extracted from BMSCs transfected with lentivirus and then was converted into cDNA. The tophat software (v2.1.0) was used to locate clean reads on the rat reference genome (RNO_6, Ensembl), allowing up to four base mismatches, and using default parameters for the rest of the parameters. The HTSeq tool (V0.6.1p2) was used to quantify gene expression based on rat genome annotation information (RNO_6) provided by Ensembl database. We analyzed the differential expression of genes by MARS (MA-plot-based method with Random Sampling model) using R’s DEGSEq software package, and then screened the significant differential expression genes between these two groups. The threshold was log2FC (normalized) > 0.585, and the *Q* value corrected by BH method was less than 0.001. The GO function enrichment and KEGG pathway analysis of differentially expressed genes were performed using DAVID (v6.8) online tools. The parameters were set as the number of enriched genes count ≥ 2, and the hypergeometric test significance threshold *p* value < 0.05 (considered as the result of significant enrichment).

### Statistical analysis

Statistical analysis was performed with the GraphPad Prism 6 statistical software package. Data are expressed as the mean ± standard deviation (SD). Differences between two groups were analyzed by independent sample *t* tests. Statistically significant differences were defined at *p < 0.05 and **p < 0.01.

## Results

### ARC promotes proliferation and inhibits apoptosis of BMSCs

**Fig. 1:**
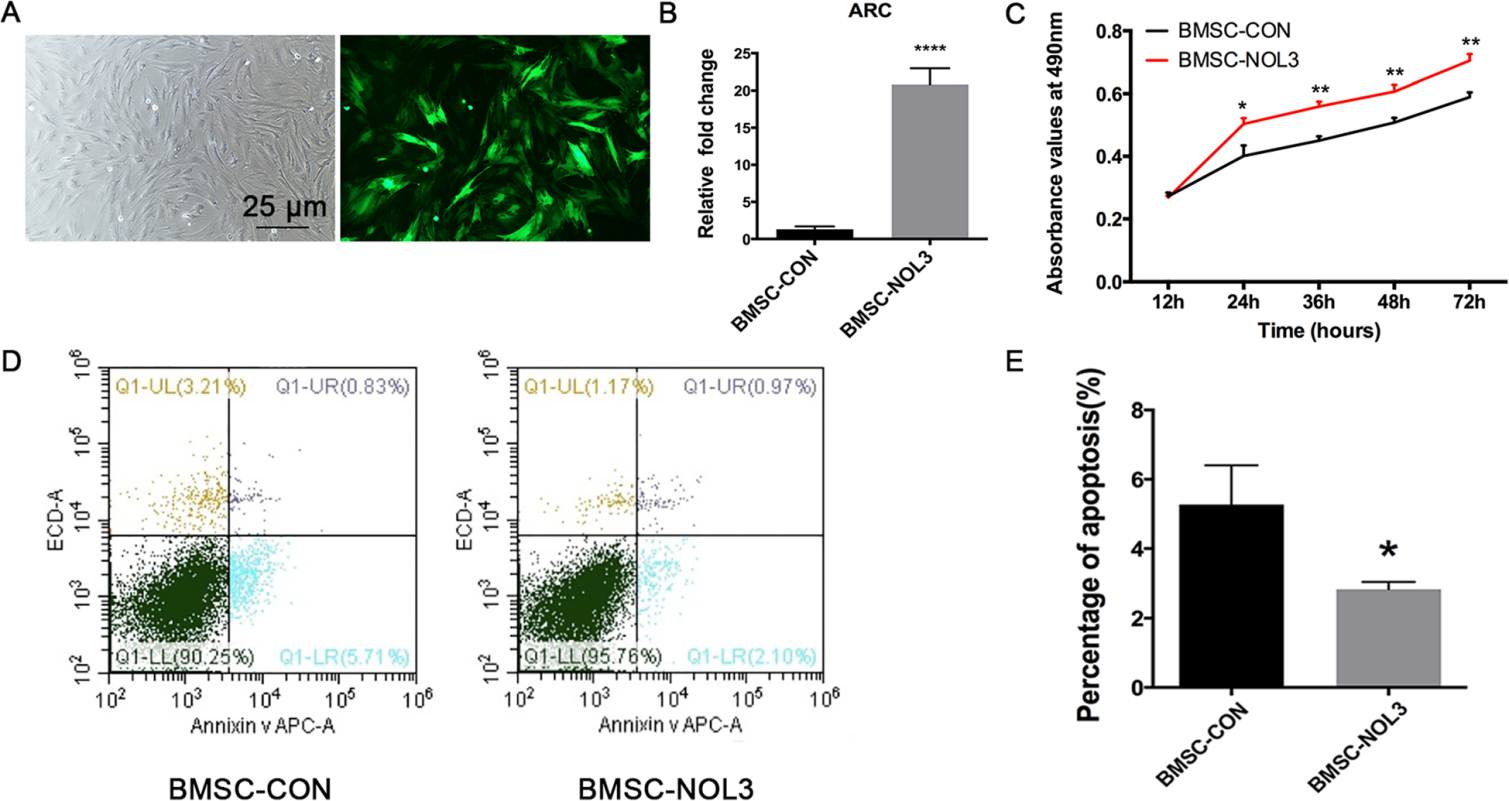
ARC overexpression analysis of BMSCs and CCK-8 and flow cytometric analyses. **a** Three days after lentivirus transduction, cells were observed under a light and fluorescence microscope. **b** ARC mRNA levels were significantly increased in the BMSC-NOL3 group compared with the BMSC-CON group (****p < 0.0001). **c** The CCK-8 method was used to detect cell proliferation from 12 h to 72 h (*p < 0.05,**p < 0.01, BMSC-CON vs. BMSC-NOL3). **d** One of three representative images of flow cytometry is presented. **e** The percentage of apoptosis was analyzed in each group (*p < 0.05)

Three days after lentivirus transduction, cells were observed under a light and fluorescence microscope (Fig. 1a), and > 70% of the BMSCs were EGFP-positive. ARC expression in the transfected BMSCs was detected by RT-PCR, as presented in Fig. 1b. ARC mRNA expression was significantly increased in the ARC-overexpressing BMSCs compared with the vector control cells. The CCK-8 method was used to detect cell numbers from 12 h to 72 h, and the ARC-overexpressing BMSCs had increased cell numbers compared with the control cells (*p < 0,05, **p < 0.01; Fig. 1c).

ARC is a highly potent and multifunctional inhibitor of apoptosis. In the current study, ARC was shown to reduce BMSC apoptosis. Cell apoptosis was detected three days after transduction, and the percentage of apoptotic cells (including the early and later stages) was 5.27% ± 0.6545% in the BMSC-CON group and 2.833% ± 0.1225% in the BMSC-NOL3 group; these values were significantly lower in the ARC-overexpressing BMSCs (Fig. 1d, e).

### ARC enhances osteogenic differentiation of BMSCs in vitro

**Fig. 2:**
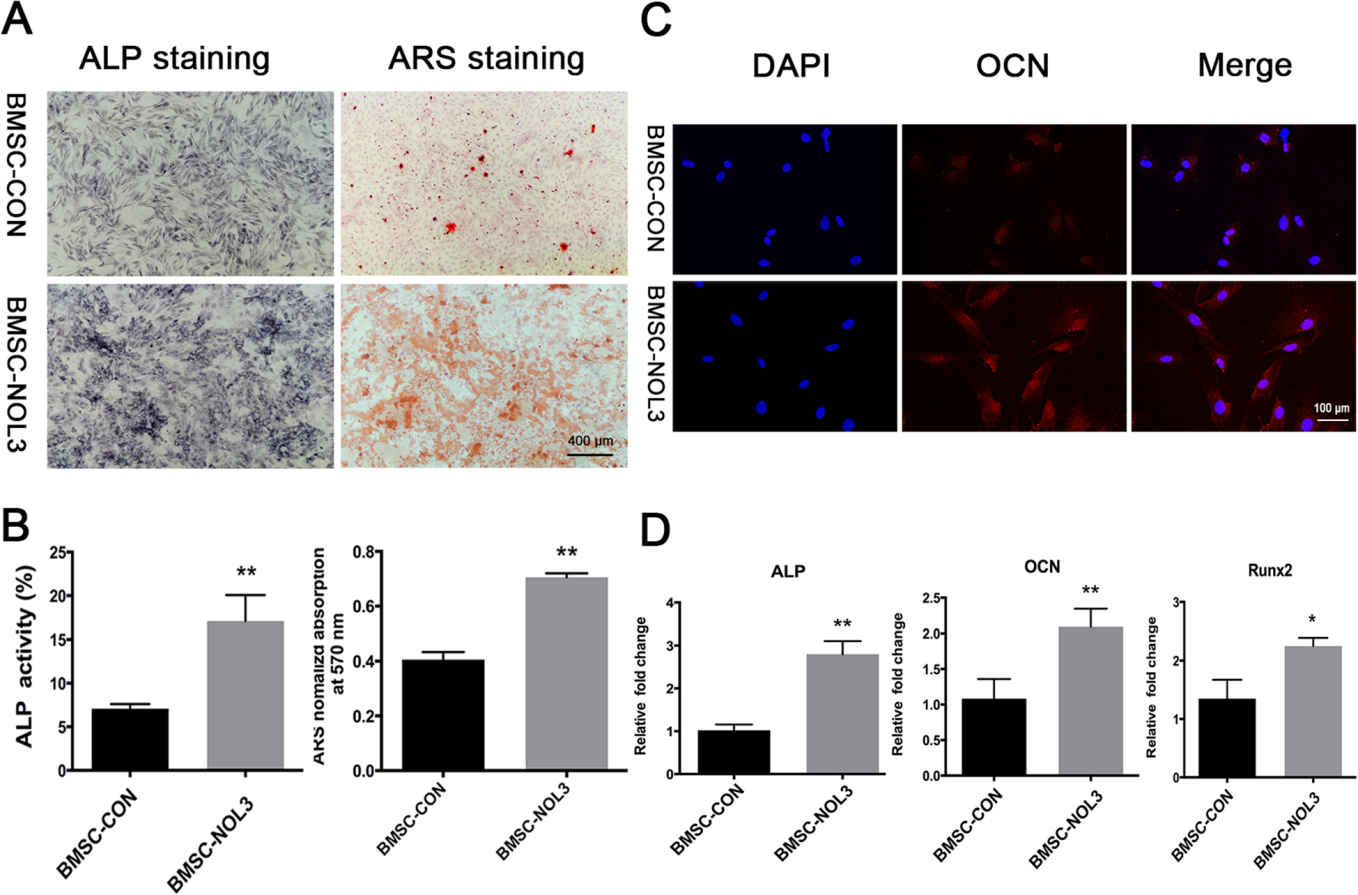
In vitro analysis of the osteogenic differentiation of the ARC-overexpressing BMSCs. **a** ALP and ARS analysis. ALP staining on day 7 and ARS staining on day 14. **b** Semiquantitative analysis of ALP activity and semiquantitative analysis of ARS (**p < 0.01). **c** Immunofluorescence staining detection of OCN protein expression. Nuclei were stained with DAPI. **d** RT-PCR analysis of osteogenic genes, including ALP, OCN, and Runx2 (*p < 0.05, **p < 0.01)

On day 7 after lentivirus transduction, ALP staining decreased in the BMSC-CON group compared with the BMSC-NOL3 group. ARS staining on day 21 revealed the same tendency as ALP staining (Fig. 2a). The semiquantitative analysis showed that ALP activity was lower in the BMSC-CON group than in the BMSC-NOL3 group. The results of the quantitative analysis of ARS were consistent with those of ALP (Fig. 2b).

Immunofluorescence staining was used to detect the expression of intracellular OCN protein. The nuclei were stained with DAPI (Fig. 2c). The results confirmed that OCN protein expression increased in the BMSC-NOL3 group compared with the BMSC-CON group.

To detect osteogenic genes, we performed RT-PCR on day 7. The levels of osteogenic genes, including ALP, OCN, and Runx2, were obviously different between these two groups. The expression of ALP, OCN, and Runx2 was significantly increased in the BMSC-NOL3 group compared with the BMSC-CON group (Fig. 2d).

All these results proved that ARC can enhance the osteogenic differentiation of BMSCs in vitro.

### Histological analysis of ectopic new bone formation and cell apoptosis detection in nude mice

**Fig. 3:**
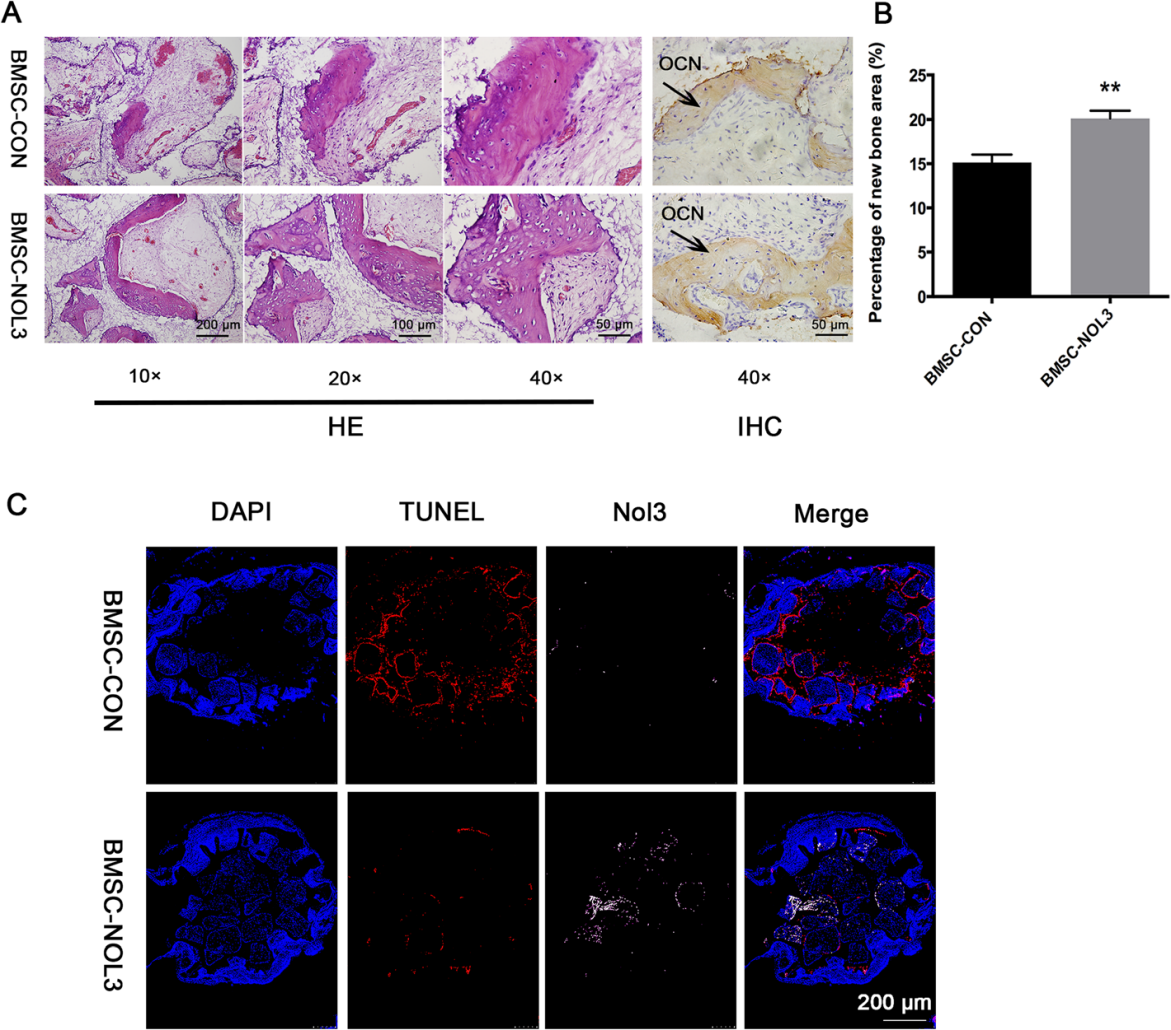
Ectopic new bone formation and cell apoptosis detection in nude mice. Cells were seeded into CPC scaffolds and then implanted subcutaneously for 8 weeks. **a** Both HE staining and OCN immunohistochemistry analysis confirmed the newly formed bone tissues in each group. **b** The percentage of new bone formation area in each group (**p < 0.01). **c** Immunofluorescence staining was carried out to detect cell apoptosis. Nuclei were stained with DAPI. Cell apoptosis was detected by a TUNEL kit and stained red. ARC protein was stained pink

Ten weeks after implantation, new bone formation was observed in each group (Fig. 3a). The percentage of new bone formation was 15.14 ± 0.3369% in the BMSC-CON group and 20.13 ± 0.3311% in the BMSC-NOL3 group. Immunohistochemical analysis of newly formed tissue was positive for the OCN protein. There was a significant difference between these two groups (**p < 0.01, Fig. 3b). These results proved that ARC can enhance new bone formation of BMSCs in vivo.

Immunofluorescence staining was carried out to detect cell apoptosis. Nuclei were stained with DAPI. Cell apoptosis was detected by a TUNEL kit and stained red. The ARC protein was stained pink. In the ARC-overexpressing group, ARC protein expression significantly increased even when the cells were implanted subcutaneously in nude mice for 8 weeks. TUNEL staining showed that cell apoptosis was significantly decreased in the ARC-overexpressing group.

### Histological, fluorochrome labeling and histomorphometric analysis of new bone regeneration

**Fig. 4:**
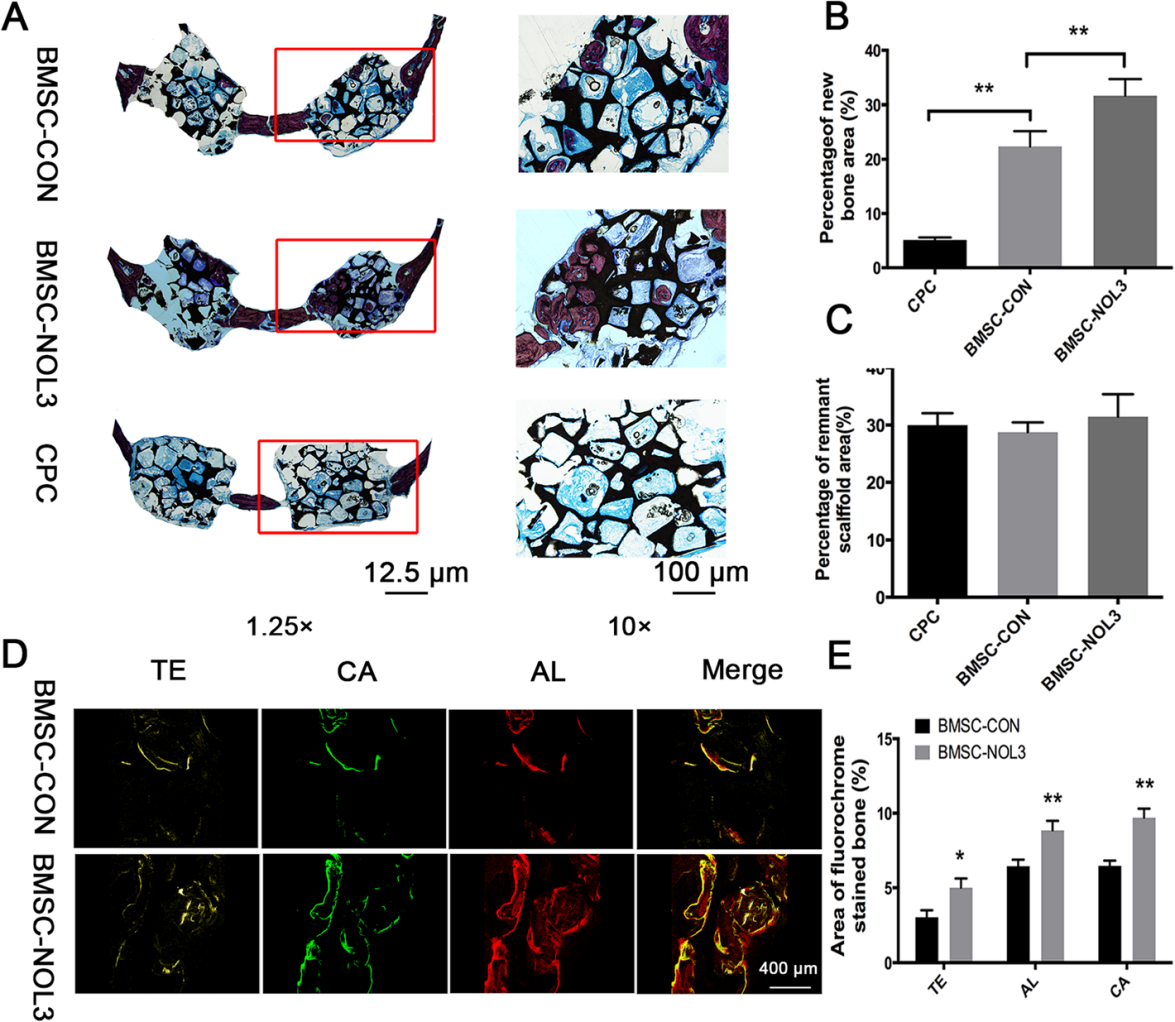
Histological analysis of the new bone formation area and remnant scaffold area in the calvarial defects. **a** The specimens were stained with Van Gieson’s picro fuchsin (original magnification, × 1.25, × 40). **b** Statistical analysis of newly formed bone tissues in each group at × 40. The percentage of new bone area was 5.135 ± 0.2263% in the CPC group, 22.36 ± 1.413% in the BMSC-CON group, and 31.64 ± 1.530% in the BMSC-NOL3 group (**p < 0.01). **c** Percentage of remnant scaffold area in each group at × 40. The percentage of remnant scaffolds was 30.00 ± 1.033% in the CPC group, 28.75 ± 0.8619% in the BMSC-CON group, and 31.46 ± 1.961% in the BMSC-NOL3 group, and no significant difference was found among these groups. **d** New bone formation and mineralization were determined by histomorphometric analysis using TE, CA, and AL fluorescent quantification, which represents the mineralization level at different time periods. **e** Percentage of the fluorochrome-stained bone area in each group

The undecalcified specimens were used to evaluate the new bone formation of BMSCs. Under light microscopy, the percentage of new bone area was 5.135 ± 0.2263% in the CPC group, 22.36 ± 1.413% in the BMSC-CON group, and 31.64 ± 1.530% in the BMSC-NOL3 group. There were significant differences between the BMSC-CON group and the CPC group and between the BMSC-CON group and the BMSC-NOL3 group (Fig. 4a, b, **p < 0.01). The percentage of remnant scaffolds was 30.00 ± 1.033% in the CPC group, 28.75 ± 0.8619% in the BMSC-CON group, and 31.46 ± 1.961% in the BMSC-NOL3 group, and no significant difference was found among these groups (Fig. 4c).

New bone formation and mineralization were determined by histomorphometric analysis by TE (tetracycline), CA (calcein), and AL (alizarin) fluorescent quantification, which represented the mineralization level at different time periods (Fig. 4d). Tetracycline hydrochloride, calcium xanthocyanin, and alizarin red indicator dyes can combine with calcium in organisms to form stable chelates, which are deposited in hard tissues and eventually form fluorescent markers to evaluate the amount of bone tissue formation. At 2 weeks, the percentage of TE labeling was 3.020 ± 0.2707% in the BMSC-CON group and 5.003 ± 0.3584% in the BMSC-NOL3 group. At 4 weeks, the percentage of CA labeling was 6.443 ± 0.2571% in the BMSC-CON group and 8.853 ± 0.3667% in the BMSC-NOL3 group. At 6 weeks, the percentage of AL labeling was 6.470 ± 0.2022% in the normoxic BMSC-CON group and 9.700 ± 0.3485% in the normoxic BMSC-NOL3 group. Statistical analysis of TE, CA, and AL fluorescence quantification showed significant differences between the BMSC-CON group and the BMSC-NOL3 group (Fig. 4e, *p < 0.05, **p < 0.01).

### ARC regulates BMSCs by activating the Fgf-2/PI3K/Akt pathway

**Fig. 5:**
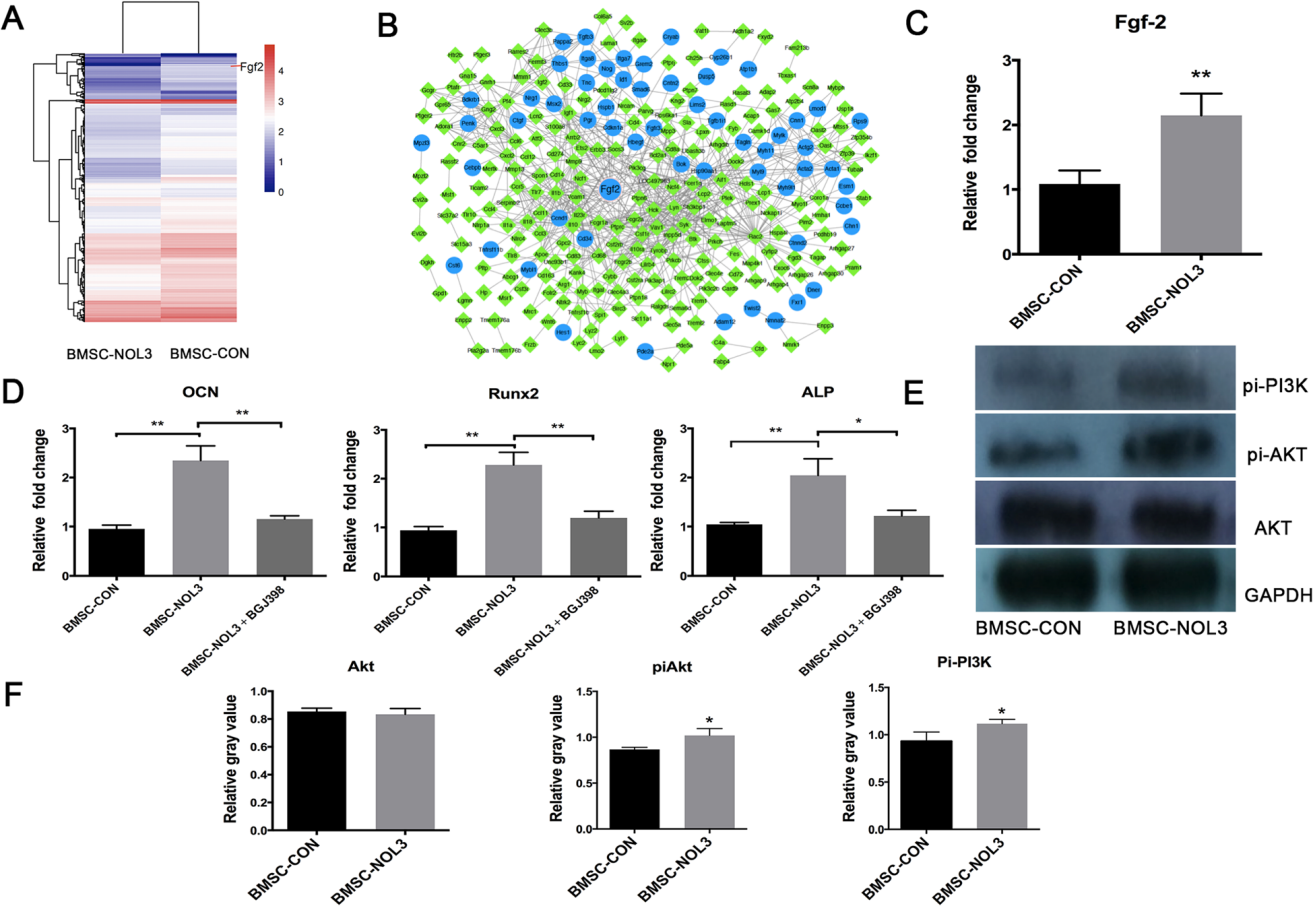
**a** Geometric mean-centered, hierarchical clustering heat map from microarray data: 533 differentially expressed genes were detected between the BMSC-CON and BMSC-NOL3 groups; 233 genes were upregulated, while 322 genes were downregulated in the BMSC-NOL3 group. **b** Differentially expressed gene-protein interaction (ppi) network diagram; the blue round node indicates upregulated gene expression, and the green diamond node indicates downregulated gene expression. **c** RT-PCR analysis of Fgf-2 gene expression between the BMSC-CON and BMSC-NOL3 groups. **d** RT-PCR analysis of osteogenic genes, including OCN, Runx2, and ALP, after BGJ398, an FGFR inhibitor, treatment. **e**, **f** WB analysis of p-PI3K and p-Akt protein expression between the BMSC-CON and BMSC-NOL3 groups

RNA-seq and bioinformatics results identified 533 differentially expressed genes between the BMSC-CON and BMSC-NOL3 groups; 233 genes were upregulated, while 322 genes were downregulated in the BMSC-NOL3 group (Fig. 5a, b). Enrichment results of the KEGG pathway analysis of differentially expressed genes showed that the TNF-α signaling pathway was inhibited in the BMSC-NOL3 group, which was in accordance with previously reported data [11]. A differentially expressed gene-protein interaction (ppi) network diagram showed that the Fgf-2 gene was upregulated in the BMSC-NOL3 group, and the RT-PCR results confirmed this finding (Fig. 5c). Fgf-2 is a stimulator of osteogenesis of human MSCs [17]. To further prove that ARC regulates BMSC osteogenic differentiation by activating the Fgf-2 signaling pathway, we added BGJ398 (0.5 μM for 2 h, from Apexbio), an FGFR inhibitor, to the transfected cells. Briefly, cells were seeded at a density of 1.0 × 10^5^ cells/well in 6-well plates and were divided into 3 groups: BMSC-CON, BMSC-NOL3, and BMSC-NOL3 cultured with BGJ398. Twenty-four hours after seeding, 0.5 μM BGJ398 was added into BMSC-NOL3 group and cultured for 2 h. TRIzol reagent (Invitrogen; Thermo Fisher Scientific, Inc.) was used to extract total cellular RNA. Then, RT-PCR was carried out to detect osteogenic genes, and the results showed that ALP, OCN and Runx2 were downregulated in the BMSC-NOL3 group after BGJ398 treatment (Fig. 5d).

FGFR can activate the PI3K/Akt signaling pathway to regulate osteogenic differentiation. In this study, the expression of p-PI3K, Akt and p-Akt was evaluated by WB. The expression of p-PI3K and p-Akt significantly increased in the BMSC-NOL3 group compared with the BMSC-CON group (Fig. 5e). Thus, ARC promotion of osteogenic differentiation of stem cells may be partially due to the activation of the Fgf-2/PI3K/Akt signaling pathway.

## Discussion

Previous studies have demonstrated the role of ARC in various cells, including cardiomyocytes, neurons, and skeletal muscle cells [18–20]. The effect of ARC on bone cells has not been reported yet except for our previously published work focusing on the effect of ARC on human osteoblasts [14]. In the present study, we explored the effect of ARC on BMSCs more deeply. In vitro and in vivo studies proved that ARC can strongly promote BMSC bone regeneration. In our study, ALP analysis, ARS staining, quantitative analysis, OCN immunofluorescence staining, and RT-PCR analysis showed that ARC can enhance BMSC osteogenic differentiation. We then created a nude mouse model and rat model of calvarium defects to evaluate the in vivo bone formation ability of the ARC-overexpressing BMSCs. The results confirmed that the ARC-overexpressing BMSCs showed increased new bone formation compared with the vector control cells.

ARC, a highly potent and multifunctional inhibitor of apoptosis, was previously shown to inhibit hypoxia-induced apoptosis in various cells. ARC functions as an antiapoptotic reagent mainly by binding proapoptotic molecules at its N-terminal CARD (caspase recruitment domain) and then further inactivating their proapoptotic functions. More recently, ARC was proven to inhibit the TNF-α pathway itself to block multiple downstream outcomes, including apoptosis, necrosis, and NF-κB activation [21]. Exogenous ARC was also shown to bind JNK and inhibit its activation in hepatocytes and islet β cells [22, 23]. In this study, the in vitro results revealed that ARC can reduce BMSC apoptosis and promote BMSC proliferation. Immunofluorescence staining of sliced tissues showed that cell apoptosis decreased in the BMSC-NOL3 group. We also detected the effect of ARC on BMSCs in a hypoxic environment in vitro, and the results confirmed that ARC can reduce hypoxia-induced apoptosis of BMSCs (data not shown). RNA-seq analysis revealed that the TNF-α signaling pathway was inhibited and the Fgf-2 gene was upregulated in the ARC-overexpressing BMSCs. RT-PCR analysis confirmed that Fgf-2 was upregulated in the BMSC-NOL3 group. Fgf-2 plays an important role in osteoblast lineage determination [24]. Alterations in Fgf-2 signaling may cause impairment of the BMSC bone formation capacity [25]. Fgf-2 activates several signaling pathways, including MEK/ERK and PI3K/AKT [26]. Fgf-2 mediated cell survival via the PI3K/AKT pathway in mouse models of neuronal differentiation, embryonic stem cells, and primary neural stem cells [27]. In this study, we examined the activation of the Fgf-2/PI3K/AKT signaling pathway in the ARC-overexpressing BMSCs. Our data indicated that activating Fgf-2/PI3-K/AKT signaling is a major mechanism through which ARC enhances BMSC-mediated new bone regeneration.

In this study, we used a lentivirus transduction method to establish ARC-overexpressing BMSCs. Gene therapy has emerged as a promising approach for repairing bone defects [28, 29], and genetically engineered cells can release growth factors at the defect area in a sustained and precise way. However, safety limits its clinical application. Protein delivery involves the application of recombinant protein in the defect area and shows potential for bone tissue engineering. In this study, we demonstrated that ARC transfected into BMSCs can powerfully enhance new bone formation. For clinical application, we further detected the effect of the ARC protein on BMSCs. Cell permeable Tat-fused NOL3 protein can be obtained for use in further studies. Generally, BMSCs are isolated from the patient and then loaded into the biomaterial scaffold along with the recombinant ARC protein and then implanted into bone defect areas, such as mandibular defects caused by trauma. Then, we further detected the effect of these implants on bone regeneration.

In conclusion, the present study suggests that ARC is a powerful agent to enhance BMSC-mediated new bone regeneration and provides a promising method for bone tissue engineering. The effect of the ARC protein on BMSCs warrants future studies.

## Data Availability

The data that support the findings of this study are available from the corresponding author upon reasonable request.

## Abbreviations

ARC: Apoptosis repressor with caspase domain
BMSCs: Bone marrow-derived mesenchymal stem cells
CPC: Calcium phosphate cement
CON: Control
FBS: Fetal bovine serum
DMEM: Dulbecco’s modified eagle medium
ALP: Alkaline phosphatase activity
ARS: Alizarin red staining
HEK293T: Human embryonic kidney 293T cells
CCK-8: Cell counting kit-8
